# fMRI Adaptation between Action Observation and Action Execution Reveals Cortical Areas with Mirror Neuron Properties in Human BA 44/45

**DOI:** 10.3389/fnhum.2016.00078

**Published:** 2016-02-29

**Authors:** Stephan de la Rosa, Frieder L. Schillinger, Heinrich H. Bülthoff, Johannes Schultz, Kamil Uludag

**Affiliations:** ^1^Department of Human Perception, Cognition and Action, Max Planck Institute for Biological CyberneticsTübingen, Germany; ^2^Georg-Elias-Müller-Institute of Psychology, University of GöttingenGöttingen, Germany; ^3^Institute of Psychology, University of GrazGraz, Austria; ^4^Medical Psychology Division, University of BonnBonn, Germany; ^5^Department of Cognitive Neuroscience, Maastricht UniversityMaastricht, Netherlands

**Keywords:** mirror neurons, fMRI, adaptation, repetition suppression, action recognition, object-directed actions, BA 44, BA 45

## Abstract

Mirror neurons (MNs) are considered to be the supporting neural mechanism for action understanding. MNs have been identified in monkey’s area F5. The identification of MNs in the human homolog of monkeys’ area F5 Broadmann Area 44/45 (BA 44/45) has been proven methodologically difficult. Cross-modal functional MRI (fMRI) adaptation studies supporting the existence of MNs restricted their analysis to *a priori* candidate regions, whereas studies that failed to find evidence used non-object-directed (NDA) actions. We tackled these limitations by using object-directed actions (ODAs) differing only in terms of their object directedness in combination with a cross-modal adaptation paradigm and a whole-brain analysis. Additionally, we tested voxels’ blood oxygenation level-dependent (BOLD) response patterns for several properties previously reported as typical MN response properties. Our results revealed 52 voxels in left inferior frontal gyrus (IFG; particularly BA 44/45), which respond to both motor and visual stimulation and exhibit cross-modal adaptation between the execution and observation of the same action. These results demonstrate that part of human IFG, specifically BA 44/45, has BOLD response characteristics very similar to monkey’s area F5.

## Introduction

Mirror neurons (MNs) are a class of neurons that respond to both the observation and the execution of a specific object-directed action (ODA). They were first recorded in area F5 of the macaque monkey (di Pellegrino et al., 1992). The response characteristic of MNs has led to the suggestion that MNs could be at the core of human action understanding by providing a close link between action and perception which allows an efficient matching of observed actions within one’s own motor system (Gallese and Goldman, 1998; Gallese et al., 2004; Rizzolatti and Sinigaglia, 2010). Therefore, MNs in humans have been considered the neural substrate that supports action understanding and action prediction.

A thorough examination of the functional role of MNs in humans requires their exact localization in the human brain. Knowing the spatial distribution of the human MN system enables researchers to investigate the activation in these cortical areas in different behavioral tasks. Accordingly, activation of MN areas in a particular behavioral task is sometimes taken as evidence for the involvement of a “mirror mechanism” in the addressed cognitive function. This approach has been taken in several studies proposing a role of MNs in cognitive functions such as imitation, empathy or theory of mind (for review, see Iacoboni, 2009). However, the conclusions based on this approach may need additional thought if the initial localization of MNs was imprecise or even incorrect.

In humans, non-invasive methods such as functional MRI (fMRI) measuring the blood oxygenation level-dependent (BOLD) signal are typically used for the identification and localization of MN areas. Many fMRI studies have used an action observation/execution or imitation task to localize MNs in humans (e.g., Iacoboni et al., 1999). In these studies, participants observed, executed and/or imitated actions and cortical areas, which were active during all three conditions, were interpreted as potential MNs sites. Based on the results of these studies several regions have been discussed as MN areas, including the ventral premotor cortex (vPM), anterior intraparietal sulcus (aIPS) and superior temporal sulcus (STS; Dinstein et al., 2007).

However, the location of MNs in humans with fMRI using this method has certain limitations (Dinstein et al., 2008). Most importantly, an activation of the same voxel during action observation and execution and/or imitation in an fMRI experiment is not indicative of the same neural population being active in all three tasks. While the same voxel being active during all three tasks is surely predicted by the presence of MNs in the voxel due to the MNs’ response characteristic (i.e., being active when an action is observed and executed), other scenarios could lead to the same result. For example, the shared activity could be owed to the activity of different neural sub-populations which are intermingled within a single voxel. In the worst case, one set of visual neurons could be active when an action is observed and a different adjacent set of neurons could be active when the same action is executed. Therefore, using common activation between action observation and execution trials for the identification of candidate cortical MNs sites need to be treated with caution.

One way to better dissociate repeated single neuron activation from the activation of different neural population on a subvoxel level is by means of fMRI adaptation (fMRI-A), which is also known as repetition suppression (RS). This method is based on the phenomenon that the BOLD signal declines if the same neural subpopulation is activated repeatedly by its preferred stimulus (Grill-Spector and Malach, 2001; Grill-Spector et al., 2006; Krekelberg et al., 2006). Because of the special response characteristics of MNs to respond to both the execution and the observation of the same action, the execution and the subsequent observation of the same action is supposed to lead to BOLD response suppression mainly in voxels containing MNs (cross-modal adaptation).

Only four studies have used fMRI-A to localize MNs in humans providing seemingly conflicting results. Studies which have found cross-modal adaptation between the execution and the observation of an action restricted their analysis to *a priori* candidate regions (Chong et al., 2008; Kilner et al., 2009), whereas studies that failed to find evidence used non-object-directed (NDA) actions (Dinstein et al., 2007; Lingnau et al., 2009). Both of these approaches to fMRI-A might constrain inferences about MNs in the human brain.

The advantage of restricting the fMRI analysis to certain candidate regions is the increase in statistical power to detect effects in these areas. On the flipside, the restriction of the fMRI analysis to candidate regions makes it difficult to get a full overview about the spatial distribution of MNs in the human brain. For instance, Kilner et al. (2009) found significant cross-modal adaptation in the left inferior frontal gyrus (IFG) and Chong et al. (2008) in the right inferior parietal lobule (IPL) using quite different tasks. Due to the restriction of the analysis to anatomically defined regions of interests (especially IFG, STS), it is difficult to say whether some of the activation found in the two studies might have actually overlapped. More importantly, the *a priori* restriction of scanning parameters to anatomically interesting regions is insensitive to other human cortical areas that also might carry MNs. For example, a recent electrophysiological study in humans identified neurons with MN properties outside previously reported human candidate MNs regions (Mukamel et al., 2010). Specifically, Mukamel et al. (2010) found neurons with MNs properties in human supplementary motor area (SMA). Moreover, a review examining the role of somatosensory cortices in social perception concluded that neurons with visuo-motor properties should also be located in somatosensory cortices (Keysers et al., 2010). Restricting the analysis to candidate cortical regions that do not comprise e.g., somatosensory areas might lead to missing additional cortical areas that also exhibit MNs response properties.

The only fMRI-A study which conducted a whole brain analysis failed to find cross-modal adaptation (Dinstein et al., 2007). In this study, participants observed and executed different symbolic hand actions (in fact they played rock-scissor-paper against a computer) and cross-modal adaptation was predicted to occur when the same action was executed and simultaneously observed (both participant and computer chose the same gesture). However, it is unclear whether such abstract symbolic hand gestures are effective in triggering MNs in humans. Indeed, studies of MNs in the macaque monkey used object-directed grasping actions as stimuli and pantomimed hand action without an object as target were less effective in triggering MNs (Umiltà et al., 2001). The other fMRI-A study that examined the locus of MNs in humans with arbitrary newly learned hand actions might have failed to find MNs activation in the human brain for a similar reason (Lingnau et al., 2009).

Overall the results of fMRI-A studies with regards to the localization of MNs in human are mixed. In sum, to date there seems to be no conclusive evidence for the candidate MNs locations in humans neither from studies using a common activation nor an fMRI-A approach to identify candidate MNs areas in humans. In particular, the difficulties of interpreting previously reported areas as candidate MNs locations arise from methodological concerns. Hence, the localization of MNs requires an approach that minimizes previous methodological concerns. The identification of potential MNs locations helps understanding the involvement of these visual-motor sensitive cortical regions in action recognition and action understanding tasks. Additionally, it is also possible to re-evaluate previously reported activations in to-be-identified motor-visual areas with regards to activation of motor-visual units.

**Figure 1:**
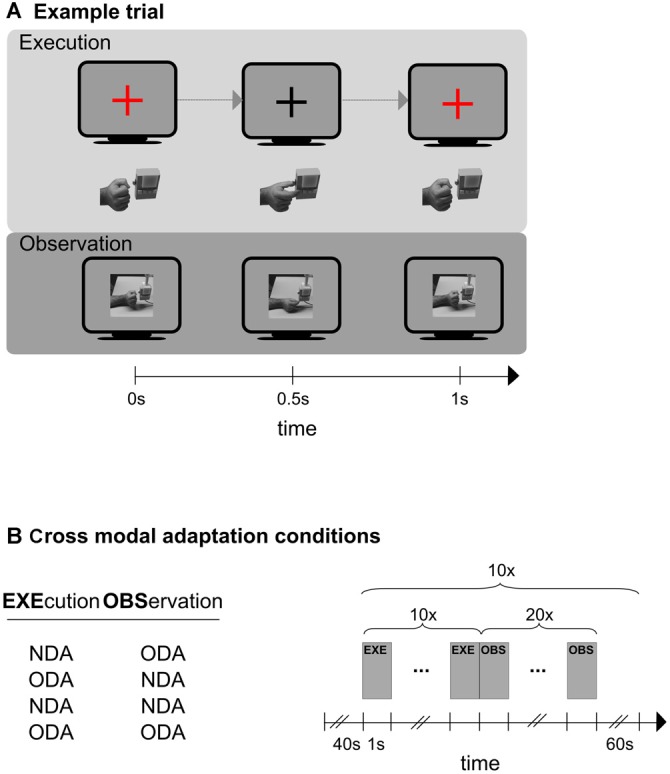
**Schematic representations of an experimental trial (A), and the experimental design of the cross modal session (B). (A)** Shown is one trial of the execution phase (top) and one trial of the observation phase (bottom). In the execution condition (top) participants synchronized their hand action (hand below monitor symbol) to the color change of a fixation cross (monitor symbol). Only the object-directed action (ODA) action is shown here. The color of the fixation cross was blue in case of an non-object-directed (NDA) action (not shown here). In the observation condition (bottom) participants observed ODA and NDA actions that were presented on the screen. Only the NDA condition is shown here. An example for an ODA is shown at the top and for the NDA at the bottom. **(B)** Left: the four cross-modal adaptation conditions. Action execution always preceded action observation. Right: schematic representation of the outline that each of the four adaptation conditions followed. A 40 s baseline preceded an experimental block in which participants first executed (EXE) an action (10 times) and then immediately afterwards observed (OBS) a action (20 times). The block was repeated 10 times. Any two blocks were separated by an inter-block-interval (IBI) of 60 s.

We designed a study that was meant to overcome the constraints of previous studies. The design of this study was inspired by findings from electrophysiological studies on MNs, namely that MNs respond to object-directed but not to non-object directed actions. Specifically, participants executed and observed a simple precision grasp towards a button box. We manipulated the “object-directedness” of the action in the following way (Figure 1A). There were two grasping actions. The object-directed action (ODA) consisted of grasping the object by moving the tips of the stretched out index finger and thumb together (resulting in a pinching grip by which the button on the button box was pressed; see Figure 1A, top). For the non-object-directed action (NDA) the same action was carried out and terminated below the button box so that the fingers tips of the index finger and thumb touched without touching the button box itself (see Figure 1A, bottom). Note, that the two actions differed in terms of their object-directedness while being associated with very similar motor actions. The purpose of the study was to first examine cortical areas selective for action execution and action observation using unimodal stimulation (unimodal session). Then we examined cross-modal adaptation transfer between action execution and action observation in a cross-modal session and a whole brain analysis. We subsequently overlapped the results of the unimodal and cross-modal session to determine motor visual areas that exhibit cross modal adaptation and therefore are associated with MNs-like BOLD response patterns.

The unimodal session meant to determine cortical regions that were sensitive to action execution and action observation of ODAs and NDAs using a whole brain analysis. To do so, participants executed and observed (in separate runs) ODAs that were alternated with NDAs while their cortical activity was recorded with fMRI. Based on the data of the unimodal session we determined the cortical areas that were sensitive to action execution and cortical areas that were sensitive to action observation.

The cross-modal session tested cortical areas for cross-modal adaptation using a whole brain analysis. To induce a cross-modal adaptation effect participants first executed (EXE) ODAs or NDAs and then observed (OBS) movies showing ODAs or NDAs. All execution phases were completely crossed with all observation phases for a total of four cross-modal adaptation conditions (remember that execution always preceded observation): ODA_EXE_/ODA_OBS_, NDA_EXE_/NDA_OBS,_ ODA_EXE_/NDA_OBS_, NDA_EXE_/ODA_OBS_ (the subscripts indicate whether the action was executed or observed). Note, that in the first two cross-modal adaptation conditions the executed action is identical to the observed action (congruent conditions); while in the latter two conditions executed and observed actions are different (incongruent conditions). Because adaptation transfer between modalities should be larger in the congruent than in the incongruent conditions, cross-modal adaptation should be stronger in the congruent than the incongruent conditions. Hence, the difference in the BOLD signal between congruent and incongruent condition serves as a measure for cross-modal adaptation.

Candidate motor-visual sites must exhibit cross-modal adaptation and should be sensitive to visual and motor stimulation. We overlapped the data of the unimodal session with that of the cross-modal sessions to determine cortical regions that were both sensitive to motor and visual stimulation and also exhibit cross-modal adaptation. Note that we did not restrict our analysis or scanning parameters to *a priori* candidate regions.

## Materials and Methods

### Participants

Ten right-handed healthy volunteers (four females) took part in the study (mean age, 22.5 years; range, 21–30 years). All subjects gave written informed consent before testing and were financially compensated for their participation in the study. The study was approved by the Ethics Review Board of the University of Tübingen and was carried out in line with the Declaration of Helsinki.

### Stimuli

There were two types of observation trials (ODA, NDA) showing two types of precision grips as videos (see Figure 1A, for examples). Both videos started with a side view of a resting male hand (fist shaped) in front of a button box. In the ODA movie, the index finger and the thumb were extended to (precision) grip the lowest button of the button box and then were moved back into the resting position. In the NDA movie, the index finger and the thumb were extended from the resting position to fake a precision grip below the button box in which the finger tips of index finger and thumb touched each other (nothing was actually gripped) and were then moved back into the resting position. The total duration of the video (1000 ms) and the time at which the grip occurred (500 ms after the start of the video) were the same in the ODA and NDA video. Participants were instructed to execute the action in synchrony with a colored fixation cross: the fixation cross appeared and stayed on for 500 ms indicating to the participants to move their hands from the resting position to the grip or non-grip position (depending on the tested condition) while the fixation cross was presented. Immediately afterwards the fixation cross turned invisible for 500 ms which indicated to participants to move their hand back into the resting position during this period. In the ODA and NDA execution trials, participants executed the same grips as in the ODA and NDA movie, respectively. To inform the participant about the type of grip to execute and to warrant the synchrony between the observed and executed grips, we used a colored fixation cross. A red fixation cross informed participants to execute an ODA and a blue fixation cross informed participants to execute an NDA. Please note that ODA and NDA actions required very similar movement patterns and had a constant spatial offset (to avoid the gripping of the box). This spatial offset was conducted by participants at the beginning of the condition. Participants received tactile feedback in both the NDA condition (from the touching of the finger tips of the index finger and thumb) and the ODA condition (from touching the object; box).

### MR Images Acquisition

Data were acquired on a 3 Tesla Siemens Trio Scanner (Siemens Medical Solutions, Erlangen, Germany) using a 12-channel birdcage head coil. Functional images were acquired with a T2*-weighted gradient-recalled echo-planar imaging (EPI) sequence with the following parameters: TR = 2500 ms; TE = 40 ms; Flip Angle = 60°; FOV = 240 × 240 × 36 mm^3^; voxel size (resolution) = 3.5 × 3.5 × 3.5 mm^3^. Thirty four slices covering the whole brain were acquired in all functional runs. The study always began with the unimodal session (for details see below) consisting of two runs of 208 volumes each (8 min 40 s). This was followed by the cross-modal session in which each participant completed 4 runs of 276 volumes each (duration of each scan: 15 min 40 s). Stimulus presentation was automatically triggered by the fMRI sequence at the beginning of each run. In order to co-register the low-resolution functional images to a high-resolution anatomical scan, a T1-weighted anatomical scan with the following parameters was acquired: TR = 1900 ms; Flip Angle = 9°; FOV = 256 × 256 × 36 mm^3^; voxel size (resolution) = 1 × 1 × 1 mm^3^. The anatomical scan comprised 176 slices (6 min 40 s) covering the whole brain and was carried out in between the second and third run of the actual experiment. The anatomical scan gave participants the opportunity to rest from the task half way through the experiment.

### Technical Setup

Participants lay supine on the scanner bed. The stimuli were back projected onto a projection screen situated behind the participant’s head and reflected into their eyes via a mirror mounted on the head coil. The projection screen was 140.5 cm from the mirror, and the stimuli subtended a maximum visual angle of approximately 9.0 ° (horizontal) × 8.3 ° (vertical). A JVC LCD projector with custom Schneider-Kreuznach long-range optics, a screen resolution of 1280 pixels × 1024 pixels and a 60 Hz refresh rate were used. The experiment was run on a 3.2 GHz Pentium 4 Windows PC with 2 GB RAM and an NVIDIA GeForce 7800 GTX graphics card with 256 MB video RAM. The program to present the stimuli and monitor the grip movements (by means of recording the button press of the button box in the ODA_EXE_ condition) was written in Matlab (Mathworks, Natick, MA, USA) using the Psychtoolbox extensions^1^ (Brainard, 1997; Pelli, 1997). Subjects’ grip actions were executed on a custom-made magnet-compatible button box. We ensured that participants executed the ODA by monitoring the signal of the response box (resulting from the key-press) and the NDA by visual inspection from the scanner control room respectively.

### Design and Procedure

At the beginning of the experiment participants received instructions about their task. This included the meaning of the colored fixation cross and how to observe and execute NDA and ODA movements. Participants always executed the movements with their right hand. For the observation trials participants were instructed to simply look at the screen and observe the displayed movie.

The experiment started with the unimodal session consisting of two types of runs each probing action observation and action execution separately (i.e., there was one observation run and one execution run). Each run started with a 40 s baseline, which consisted of a gray blank screen during which the participant rested. Sixteen blocks of observation or execution trials (depending on the type of run) immediately followed this baseline. A block of trials (10 trials) probed always the same movement type (either ODA or NDA). Each trial within a block had a 1 s duration. Trials were immediately following each other. There were eight ODA and eight NDA blocks, which alternated within each run to a total of 16 blocks per run. Blocks were separated by a 20 s inter block interval (IBI). Hence, the total run length was 8 min 40 s. The testing order of the execution and observation runs was counterbalanced across participants. The data of the first session was used to determine cortical areas that respond to both visual and motor stimulation.

The cross modal sessions followed the unimodal session. Here, participants were tested on four cross-modal adaptation conditions to identify cortical regions exhibiting cross-modal transfer. Specifically, each cross-modal adaptation condition probed a different execution-observation transition, namely ODA_EXE_/ODA_OBS_, NDA_EXE_/NDA_OBS,_ ODA_EXE_/NDA_OBS_, NDA_EXE_/ODA_OBS_ (Figure 1B, left panel). Participants were informed by the experimenter about the upcoming cross-modal adaptation condition before the condition started. The testing order of four cross-modal adaptation conditions was randomized across participants. Each condition started with a blank screen of 40 s (baseline) and 10 presentations of the following sequence: ten execution trials (each 1 s), which were immediately followed by 20 observation trials (each 1 s), and a blank screen presented for 60 s (in this order; Figure 1B, right panel). The number of observation trials was chosen to 20 to ensure that interval of interest was sufficiently long to capture the adaptation response (which would occur in the observation interval). In total, this amounted to a length of 15 min 40 s for each condition. The testing order of these four conditions was randomized for each participant.

### fMRI Data Analysis

#### Data Pre-Processing

The data was analyzed using the FSL program package (Version 4.0, Analysis Group, FMRIB, Oxford, UK; Smith et al., 2004). All functional runs were motion-corrected to the middle volume of each individual run using the *MCFLIRT* feature of FSL (Jenkinson and Smith, 2001). Preprocessing of the data was completed with *FEAT* (Woolrich et al., 2001) and included a high-pass filtering of 100 Hz to detrend the data, spatial smoothing (5 mm), and prewhitening (Woolrich et al., 2001). Gray matter contour was extracted from the anatomical high resolution scans applying the *BET* routine of FSL (Smith, 2002). Subject’s structural images were spatially normalized using the MNI 152 average brain. Functional runs of each participant were spatially registered to the corresponding brain-extracted image.

#### Data Analysis

Statistical maps were generated with *FEAT*, performing a general linear model (GLM) analysis. For sake of clarity, we present a brief overview of our analysis, which aimed at identifying cortical regions exhibiting several important motor-visual properties (visual sensitivity, motor sensitivity, cross modal adaptation) as measured by BOLD response. Note, that we only consider the resulting cortical areas of step 3 as candidate motor-visual areas. We will explain each step and the rationale for it in detail later:

Step 1. Testing for motor-visual sensitivity in the whole brain using the data of the unimodal session: the contrasts observation > baseline and execution > baseline were calculated separately on the data from the two unimodal runs.
Step 2. Testing for cross-modal adaptation (AD) only within the observation phase using the data of the cross-modal session (see Equation 1 below).
Step 3. Determining cortical regions that survived all three contrasts outlined in steps 1 and 2.

Step 1: We used the data of the unimodal session to determine cortical areas that respond to both observation and execution by calculating the contrast observation > baseline and execution > baseline (step 1). In the calculation of these contrasts the data was collapsed across ODAs and NDAs to gain more statistical power. These two contrasts were calculated separately in a brain-wise fashion for each participant using fixed effects GLMs. On the group level we used FSL’s FLAME 1 + 2 (mixed effects model) analysis. We controlled the family wise error rate by using a cluster-forming *z*-threshold of 2.3 and a [Gaussian Random Field (GRF) Theory corrected] cluster significance threshold of *p* = 0.05 for each contrast.

Step 2: In step 2, we calculated the cross-modal adaptation effect. Because the cross-modal adaptation effect occurs only after switching modalities from execution to observation, we expected the cross modal adaptation effect to occur during the observation phase. Hence, our calculations of the adaptation effect were based on the data of the observation phase only in the cross modal session. Specifically, the adaptation difference (AD) was calculated for each participant in the following way:

(1) $$\begin{array}{l} AD\;=({\text{NDA}}_{\text{EXE}}/{\text{ODA}}_{\text{OBS}}\;+{\text{ODA}}_{\text{EXE}}/{\text{NDA}}_{\text{OBS}})\\ \;-\;({\text{NDA}}_{\text{EXE}}{\text{/NDA}}_{\text{OBS}}{\text{+ODA}}_{\text{EXE}}{\text{/ODA}}_{\text{OBS}}) \end{array}$$

Note, the two subtrahends only differ in terms of their congruency. That is, the first subtrahend consists of conditions in which the executed and observed grips were different (incongruent conditions) while the second subtrahend consists of conditions in which the executed and observed grips were the same (congruent conditions). Hence, AD measures the BOLD signal difference between incongruent conditions, for which only little cross-modal adaptation is expected in the observation phase, and congruent conditions, for which larger cross-modal adaptation is expected in the observation phase. Due to the expected larger adaptation effect in the congruent conditions, the BOLD response should decline more in the congruent than incongruent conditions during the observation phase. Consequently, an AD value larger than zero would be indicative of cross-modal adaptation because congruent conditions (which should have a smaller BOLD response in the observation phase) are subtracted from incongruent conditions (which should be associated with a larger BOLD response in the observation phase). The AD contrast was calculated for each participant separately using a fixed effect GLM. The group level analysis for the AD contrast was conducted using FLAME 1 + 2. We protected against family-wise false positives using a cluster-forming *z*-threshold of 2.3 and a (GRF corrected) cluster significance threshold of *p* = 0.05.

Step 3: We then looked for cortical regions that survived all three contrasts (observation > baseline, execution > baseline, and the AD contrast) by overlaying the resulting cluster maps of the three steps.

Finally, we visually inspected the time courses of the resulting cluster of step 3. We created a mask of the voxels that survived all previous contrasts (52 voxels) in the standard space. This mask was registered with the 4D functional brain image by means of FSL command *FLIRT* for each participant and condition separately. We then extracted the average time series of the raw BOLD signal for the 52 voxels for each participant and condition. The BOLD response was centered for each participant and condition separately relative to the baseline condition.

## Results

### Step 1: Motor and Visual Sensitivity

**Figure 2:**
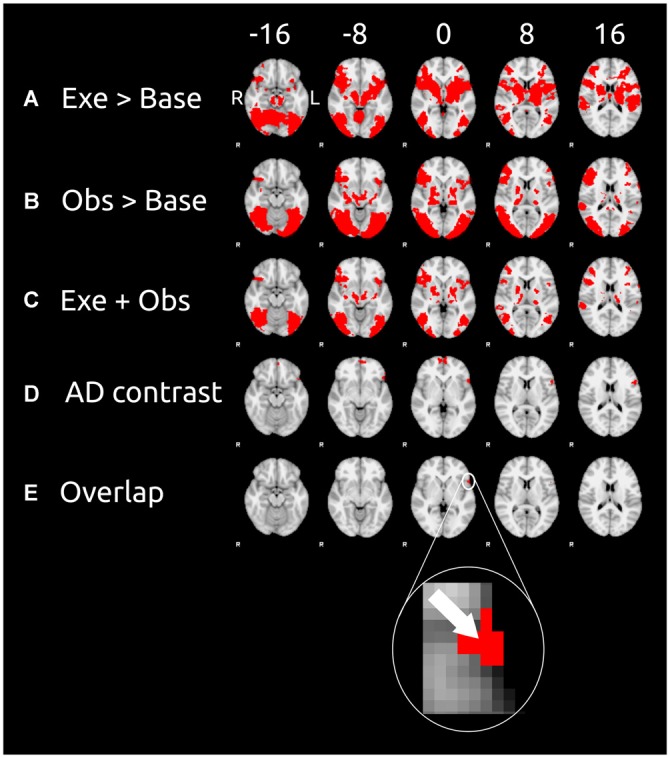
**Cortical clusters exhibiting significant activation as revealed by the contrasts (left) of the unimodal session and cross-modal session.** These contrasts were calculated on the data of the 10 participants. Six equidistant transverse slices (exact coordinates are shown at the top) were taken for each contrast of our analysis (contrast labels are in the leftmost column). **(A)** The results of the contrast execution > baseline (unimodal session; results of step 1); **(B)** the results of the contrast observation > baseline (unimodal session; results of step 1); **(C)** cortical areas that survived both of the previous contrasts; **(D)** the results of the cross-modal adaptation contrast (cross-modal session; results of step 2) AD is the adaptation difference (AD) as defined in Equation (1); **(E)** cortical areas that survived all three previous contrasts (results of step 3). For sake of clarity the arrow in inset shows the voxel with the peak activity (as measured by the AD contrast) in the overlap cluster. Data are displayed in the radiological convention: left hemisphere on the right of each slice.

Figures 2A,B show the results of unimodal contrasts (execution > baseline and observation > baseline, respectively). Each of the contrast was calculated on the whole brain. Figure 2A shows the results of the contrast execution > baseline. Action execution causes wide spread cortical activation mainly in the occipital cortex, right anterior intra-parietal sulcus, IPL, somatosensory cortex, primary motor cortex, and IFG, and supplementary motor cortex. Action observation causes large activation in occipital cortex (more widespread than for action execution), anterior intra-parietal sulcus, inferior partietal lobule, IFG, and supplementary motor cortex (see Figure 2B which shows the contrast observation > baseline). Common motor-visual activations (Figure 2C) are found in visual cortex, occipital cortex, right anterior intra-parietal sulcus, IPL, IFG, supplementary motor cortex, and frontal pole.

### Step 2: Cortical Areas of Cross Modal Adaptation

The AD contrast was calculated on the data of the observation phase only because we expected cross modal to only occur after the switch of modalities, i.e., in the observation but not the execution phase. Cortical areas sensitive to cross modal adaptation were found in the frontal cortical areas (see Figure 2D), namely frontal pole and the IFG.

### Step 3: Motor Visual Areas Showing Cross-Modal Adaptation

We defined motor-visual cortical areas as cortical areas that survived all three previous contrasts (i.e., execution > baseline, observation > baseline, and AD contrast). The union of these three contrast is shown in Figure 2E. Only one cluster located in the left IFG survived all three contrasts. This cluster consisted of 52 voxels and had its peak activity at the *X* = −54, *Y* = 18, *Z* = 0 (MNI coordinates). According to the Juelich Histological Atlas the peak voxel (*z* = 3.36 for the adaptation contrast) has a probability of 46% to be associated with Broadmann Area 44 (BA 44) and 38% probability to be associated with of BA 45. These 52 voxels of this analysis in BA 44/45 have typical response properties of motor visual units: they respond to both visual and motor stimulation and show cross-modal adaptation transfer.

**Figure 3:**
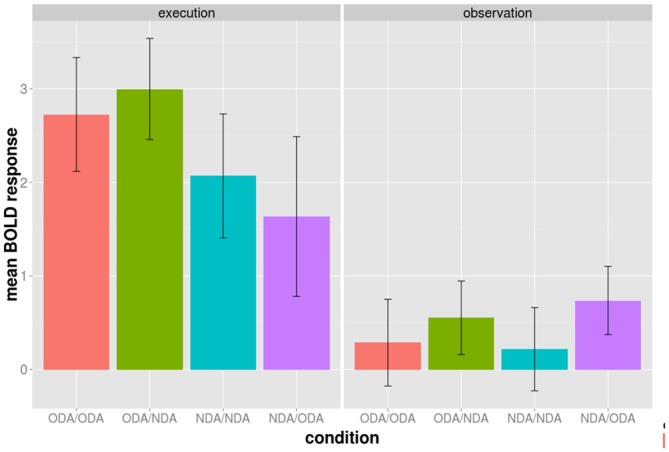
**Average BOLD activation of the 52 BA 44/45 voxels during the execution (left panel) and observation phase (right panel) shown for each of the four experimental conditions of the cross-modal session separately.** The conditions are color grouped according to the type of action movement during the execution phase. Bars indicated one standard error from the mean.

We examined whether the adaptation effect was only driven by either the ODA or the NDA alone in above regions during the observation phase. We calculated the mean BOLD response during the observation phase from the 52 voxels for each condition separately (Figure 3). Both the ODA/ODA and the NDA/NDA condition have a lower BOLD response than the ODA/NDA and the NDA/ODA condition, respectively. More specifically, the difference ODA/NDA − ODA/ODA was not statistically significantly different from the difference NDA/ODA − NDA/NDA, *t*_(9)_ = 0.324, *d* = 0.102, *p* = 0.7533. This result is in line with the idea that both congruent NDA and ODA conditions contributed to the adaptation effect.

### Time Course of the BOLD Response

**Figure 4:**
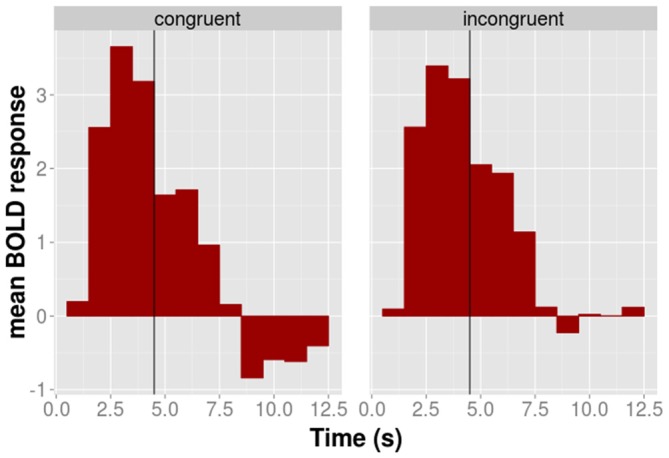
**The time course of the average BOLD response of the 52 BA 44/45 voxels shown for congruent (left panel) and incongruent (right panel) conditions separately.** The black vertical line indicates the transition between the execution and the observation phase.

The time course of the adaptation effect within the 52 voxels is shown for illustrative purposes in Figure 4. During the motor phase the BOLD signal of the congruent condition is slightly above that of the incongruent conditions. This pattern reverses during the observation phase in which the activation of the congruent condition lies below that of the incongruent condition. The AD contrast, which was calculated on the observation phase only, suggested that the difference between congruent and incongruent conditions during the observation was significant.

Finally, we were interested in whether congruent and incongruent conditions differ during the execution phase. We computed the difference between congruent and incongruent conditions on the data of the execution phase only. This contrast was calculated on the whole brain in analogy to AD contrast calculation of step 2. The results showed no significant activation in the 52 voxel for the congruent > incongruent contrast. Hence, congruent and incongruent condition were not associated with statistically significantly different BOLD responses during the execution phase.

## Discussion

The aim of the present study was to provide evidence for the location of motor-visual units in humans using an fMRI design that attempted to minimize previously identified methodological constraints. We aimed at minimizing the effect of *a priori* assumptions about the location of motor-visual units by conducting a whole-brain analysis. We characterized motor-visual areas in humans by looking for cortical areas that are associated with BOLD response properties that have been previously reported with MNs. Specifically, we combined a cross-modal adaptation paradigm using actions that differed mainly in their object-directedness with a whole-brain analysis for the identification of motor-visual areas. We further checked these voxels for their sensitivity to motor and visual stimulation. In the end, 52 voxels within the IFG exhibited all these characteristics and, therefore, are associated with BOLD response properties that resemble those of MNs. The specific location of these voxels is associated with BA 44 and BA 45. Importantly BA 44/45 is a candidate area in humans that is believed to be homologs to monkey area F5 (Petrides, 2005). We, therefore, show that the modulation of the BOLD response in this area is in line with what is expected from motor-visual units.

The left IFG has previously been shown to be activated during the observation and the execution of the same action (Iacoboni et al., 1999; Nishitani and Hari, 2000; Buccino et al., 2001; Gazzola and Keysers, 2009). For example, Iacoboni et al. (1999) found activation in BA 44 in a finger tapping imitation fMRI study both when finger actions were executed and observed. Similarly, Nishitani and Hari (2000) found in an MEG study that the left BA 44 is strongly activated by action imitation for a pinching action compared to other non-object directed actions. Moreover, the temporal characteristics of this area led Nishitani and Hari (2000) to suggest that IFG is central to the functioning of the MN system. Overall, left IFG and region BA 44 appears to be candidate site for MNs. Our results confirm this observation by showing that the IFG (BA 44/45 specifically) shows cross-modal adaptation transfer, and are sensitive to motor and visual stimulation.

The small number of motor-visual voxels found in this study is not entirely surprising given that physiological studies found that only 25% of the tested neurons exhibit MN properties (Rizzolatti and Craighero, 2004).

Because participants were right-handed and always executed the action with their right hand, we cannot exclude the possibility that the left hemispheric activation for the adaptation effect observed in this experiment was owed to this right-handed bias.

Our results are similar to previous findings from Kilner et al.’s (2009) fMRI-A study. They also found cross-modal activation in the IFG, however, their activation was slightly more dorsal and posterior to the IFG activation found in the present study. For sake of clarity their peak activation (MNI coordinates: −50, −2, 12) is associated with a 37% probability of being located in the secondary somatosensory cortex and 10% in BA 44, while our peak activation (−54, 18, 0) had probabilities of 46% for BA 44 and 38% for BA 45. This difference in localization might be explained by the different kind of stimuli that were used in the experiments. While Kilner et al. (2009) contrasted the activity associated with two different kinds of object directed actions, the present study contrasted object and non-object directed actions.

In contrast, other fMRI-A studies have failed to find activation in the IFG (Dinstein et al., 2007; Chong et al., 2008; Lingnau et al., 2009) using a fMRI-A paradigm. What might be possible reasons? The two studies that found cross modal adaptation in IFG (the present study and Kilner et al., 2009) using fMRI-A explicitly employed object directed actions for the action observation and action execution trials. In contrast, the other studies that did not find an fMRI-A adaptation effect in IFG used non-object directed actions for their motor and visual stimulation. Specifically, Dinstein et al. (2007) used rock-paper-scissors game hand gestures, Chong et al. (2008) used pantomimed hand gestures (e.g., shoot gun), and Lingnau et al. (2009) used meaningless hand gestures. One major difference between studies finding cross-modal adaptation in the IFG and those not finding it is the use of object directed actions. Kilner et al. (2009) also discussed optimized scan parameters as another possible reason for the lack of finding cross-modal adaptation in the three previously mentioned studies. Specifically, Kilner et al. (2009) suggested that the optimization of the scanning parameters to capture activation in the IFG region in their study might have led to a more powerful design in comparison to non-restricted analysis. Our study contributes to this discussion by showing that IFG activity is found within a much less restricted analysis suggesting that optimization of the scan parameters alone is not the sole reason for finding cross modal adaptation in IFG. Rather our results speak to IFG activation being caused by the presence of transitive actions.

The location of the BA 44/45 area in our study is somewhat more ventral than for activations typically found with observation of actions (for a review, see Van Overwalle and Baetens, 2009). Although this might seem surprising at first, it is important to remember that not all areas of action observation constitute MNs areas. Hence, one cannot readily expect to observe cross-modal adaptation in these areas. Moreover, the BA 44/45 location reported in this study has been previously associated with sensitivity to motor and visual stimulation. For example, Fridman et al. (2006) report the ventral part of BA 44/45 to be activated during action execution. Likewise, this cortical ares is also activated during the verbalization and reading of words (Herbster et al., 1997; Tan et al., 2001; Abrahams et al., 2003) and the passive viewing of words (Menard et al., 1996), which had led to the suggestion that symbolic semantics are grounded in perception and action systems (Pulvermüller and Fadiga, 2010; Pulvermüller, 2013). Taken together, the ventral part of BA 44/45 has been associated with activation during observation and execution, which is in line with our observation.

Mukamel et al. (2010) found neural units that responded to motor and visual stimulation in the human SMA. Here, we report cross-modal activation in the IFG and more specifically BA 44/45. Although these results might appear contradictory at first, note that we also find the bilateral SMA to be significantly activated during action execution and action observation. Our results, therefore, replicate previous reports about the visual and motor sensitivity of SMA. However, we did not observe an adaptation effect in this region. One reason could be that single cell recordings and whole brain analyses are associated with different sensitivities to detect changes at the neuronal level. Alternatively, it is possible that not all neural units exhibit adaptation effects. Hence, testing for cross-modal adaptation might account for the differences between Mukamel et al. (2010) and our results. At this point, we can only speculate about the lack of adaptation effects in SMA.

It is important to note that cross-modal adaptation might occur in other cortical regions apart from the 52 voxels in BA 44/45 reported in this study. The lack of evidence for cross-modal adaptation in other cortical areas is not the evidence for the lack of cross-modal units in these areas. In other words, the absence of cross-modal evidence in other cortical areas might be simply a matter of a lack in statistical power. In this light, our results are indicative of cortical areas that show the strongest cross-modal adaptation within our experimental paradigm.

What is the physiological basis of the adaptation effect? Recent evidence demonstrated that the neural firing of MNs is unaffected by the repeated visual presentation of an action (Caggiano et al., 2013). However, the same study found a change in the local field potential (LFP) due to the repeated observation of an action. Since the BOLD signal correlates better with changes of LFP than with changes of neural firing (Logothetis et al., 2001; Ekstrom et al., 2009), we suggest that the observed adaptation effect reflects changes in the LFP rather than a change of neural firing of MNs.

Supporting evidence for the role of IFG in action production and action recognition comes from clinical lesion studies with apraxic and aphasic patients. Goldenberg et al. (2007) showed that deficits in pantomime tool use in apraxic patients is correlated with damages to the left IFG. In the same vein, Saygin et al. (2004) provided evidence that the impaired recognition of action images but not of action words are linked to damages in IFG. These lesion studies therefore support the idea that IFG (of which BA 44/45 is a part) is implicated in action execution and observation. However, future research needs to determine whether motor-visual areas identified in this study contribute to social cognition.

In the current study, we tested grasp vs. non-grasp actions because we wanted to ensure that the kinematic patterns during action observation and execution are as similar as possible. As a result, we did not probe different object direction actions (e.g., button press vs. tapping of a box). Although the 52 voxels in IFG are selective for ODAs it is not yet clear the degree to which these voxels are selective for different ODAs. Future research is necessary to determine to what degree the cortical area reported in this study is selective for different ODAs.

In summary, we find 52 voxels in IFG and more specifically in BA 44/45 whose response to a visual object directed action changes depending on the object-directedness of an immediately preceding action execution. Moreover, these voxels are sensitive to both visual and motor stimulation. These voxels, therefore, are interesting for the discussion of the location of MNs.

## Author Contributions

SDLR and KU designed the study; SDLR and FLS prepared the study; FLS collected the data; SDLR, JS, and FLS analyzed the results; all authors wrote the article.

## Funding

The contribution of SDLR was funded by the EU Project TANGO (ICT-2009-C 249858).

## Conflict of Interest Statement

The authors declare that the research was conducted in the absence of any commercial or financial relationships that could be construed as a potential conflict of interest.

## Footnotes

^1^http://www.psychtoolbox.org
